# Improving Orientation to Psychiatric Inpatient Admission Through a Co-Produced Video: A Quality Improvement Project

**DOI:** 10.1192/bjo.2026.11487

**Published:** 2026-06-30

**Authors:** Claire Palmer, Mitashi Singh

**Affiliations:** 1 Oxford Health NHS Foundation Trust, Aylesbury, United Kingdom; 2 Oxford Health NHS Foundation Trust, Oxford, United Kingdom

## Abstract

**Aims:**

A psychiatric inpatient admission can be the most challenging part of navigating mental illness. Patients are often at their most vulnerable when facing the unknowns of an admission. Patients are usually oriented to the wards through conversation, a quick tour or written material provided at the time of admission. These existing interventions vary in delivery and uptake and standardised, accessible orientation materials remain scarce. This quality improvement project (QIP) evaluated the impact of a co-produced animated orientation video on patients’ perceived knowledge and anxiety around admission. Secondary aims included exploring experiences of existing orientation interventions, identifying anxiety sources and knowledge gaps, and gathering feedback.

**Methods:**

This mixed-methods QIP was conducted at two acute mental health wards for adults at the Whiteleaf Centre, Aylesbury. An animated orientation video was co-produced by the in-patient transformation team, clinicians, and experts-by-experience. A brief questionnaire was designed with inputs from service users and administered to patients admitted between July and November. Pre-determined exclusion criteria were: declined participation, in seclusion, not oriented, and >48 hours since admission. Patients rated knowledge and anxiety on single Likert items pre- and post-video, and answered open-ended questions about anxiety sources, knowledge gaps, existing interventions, and video feedback. Quantitative data were analysed using Wilcoxon signed-rank tests.

**Results:**

Of 31 patients approached, 20 were included (14 male, 6 female; age range 17–63; mean 36.1 years); 80% were experiencing their first admission. At baseline, 55% reported anxiety and 35% reported that they did not know what to expect upon admission. Helpfulness of existing interventions varied: ward tour 82% (14/17), one-to-one chat 86% (12/14), patientinformation pack 50% (8/16). 85% (17/20) found the video helpful. Knowledge improved significantly (median 3.0 to 4.0; W=55.0, p=0.001); anxiety showed a favourable but non-significant trend (median 4.0 to 3.0; W=6.0, p=0.125), with no patient reporting increased anxiety. Themes that emerged highlighted reassurance, clarity about facilities, and structured orientation as strengths. Suggestions included adding content on leave arrangements, treatment types, and ward-specific information. The animated format was highlighted as a strength by some patients (2/20) and a distractor by others (3/20).

**Conclusion:**

A co-produced orientation video is an acceptable and effective intervention for improving knowledge of what to expect during psychiatric inpatient admission. Iterative refinement of video design and content can further tailor it to meet patients' needs.

